# An insightful estimation of undergraduate medical students’ experience about the Flipped Classroom

**DOI:** 10.12669/pjms.38.1.4256

**Published:** 2022

**Authors:** Hamza Mohammad Abdulghani, Kamran Sattar, Tauseef Ahmad, Dost Muhammad Halepoto, Ashfaq Akram

**Affiliations:** 1 Hamza Mohammad Abdulghani Department of Medical Education, College of Medicine, King Saud University, Riyadh, Saudi Arabia; 2 Kamran Sattar Department of Medical Education, Department of Medical Education, School of Medical Sciences, Universiti Sains Malaysia, Kelantan, Malaysia. College of Medicine, King Saud University, Riyadh, Saudi Arabia; 3 Tauseef Ahmad Department of Medical Education, Department of Computer Science and Engineering, NIMS University, Jaipur, Rajasthan, India. College of Medicine, King Saud University, Riyadh, Saudi Arabia; 4 Dost Muhammad Halepoto Department of Physiology, College of Medicine, King Saud University, Riyadh, Saudi Arabia; 5 Ashfaq Akram Department of Medical Education, College of Medicine, King Saud University, Riyadh, Saudi Arabia

**Keywords:** Flipped Classroom, Online learning, Undergraduate, Medical Student

## Abstract

**Background and Objective:**

The Flipped Classroom (FC) approach has become increasingly predominant and popular in medical education. This study aimed to explore the usefulness and the scope of FC based on medical students’ experience, with their adaptation challenges.

**Methods:**

The present study was a mixed-method accomplished during the academic years 2019-20, involving fourth-year students at the College of Medicine in Riyadh, Saudi Arabia. A self-administered questionnaire was used to seek their first experience and opinion of the FC.

**Results:**

A total of 234 questionnaires were distributed to the students, and 214 students completed the survey (response rate of 91.45%). Out of this total, 68.2 % were males and 31.8% were females. Most of the students agreed 156 (72.9%) that the flipped classroom was more engaging than the traditional lecture, among them 100 (68.5%) males and 56 (82.3) females agreed. Almost ~79% of students liked FC as it enabled them knowing the material in advance, and the class time was spent clarifying the facts and principles with active interaction, as commented during focus group discussion *“More chance for discussing with the doctors, and I got the chance to answer”* (St. 6).

**Conclusion:**

The results showed that the students like the FC more than the conventional classroom. Suggestions were given by students to improve the active learning sessions within the FC modality.

## INTRODUCTION

Flipped classroom (FC) is a mixed learning modality where students learn partly at a controlled face-to-face campus location, and partly via the internet. The use of the FC approach has become progressively prevalent in medical education.^1^ Medical educationists have been continually/progressively changing the instructional modality, decreasing the number of lectures, using online technology and adding self-directed learning; and encouraging inter-professional education.^2^,^3^ Among many learning styles, strategies, and approaches, the FC (also called inverted classrooms) have generated considerable popularity.^4^,^5^ Hence it is embraced in various health professional curricula. In the digital era, learners can access the prescribed content easier than ever before, rendering self-regulated learning aligned with learning outcomes.^6^

In FC, before class, students are provided with foundational content, allowing them to come to the class with a better knowledge base. They can note down questions while watching video discourses, podcasts, PowerPoints, or the use of any other online learning resources as advised by their instructors. Many educational videos available on the internet can be used by faculty members who flip their classrooms. This allows for an early encounter with the learning material. Lecture substances are moved outside the classroom, permitting more training and discussion inside the classroom. Contrary to the traditional class environment, it lets students have a more interactive setting.^7^

This dynamic transformation through FC lets students play an active role in achieving the learning objectives.^8^ However, the literature and adaptation of FC in Saudi medical education system are in an infancy stage. This study aimed to assess the effectiveness of FC, and explore of medical students’ experiences, with their adapted learning environment during pandemic COVID-19.

## METHODS

### Structure of Flipped Classrooms:

**Fig.1:**
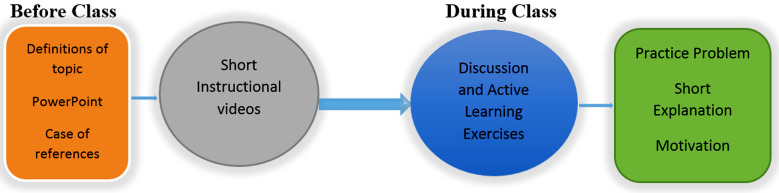
Flow-chart of the Flipped Classroom process model.

Teaching-learning activities outside the classroom were structured and informed to the 4^th^ year medical students. The provision of recorded lectures followed by in-class exercises (Fig.1) model of FC was adopted. Thus, the PowerPoint slide, subject notes or homework, short videos (no more than 10 to 20 minutes), and guiding questions to identify the course objectives were prepared. The tutors (as on-site experts) clarified the content and monitored the progress where students had learned about the subject outside the class. The workgroups of students were established to resolve a problem if it may happen (Fig.1).

### Context:

The family medicine course was introduced at College of Medicine, King Saud University during the academic year 2019-20, to achieve essential competencies listed in the Saudi Medical Framework. This course comprised 20 interactive sessions (each session 1-hour duration). However, the FC was adopted for three topics (headache, hypertension, diabetes mellitus), introduced in the family medicine course. This course had four credit hours, and a total of 13 lecture sessions. Students were informed to read the above three topics and watch related videos available on a blog prepared by the tutors. This study was approved by the Institutional Review Board and consents were obtained before the data collection.

### Study Design:

This was a mixed-mode study, carried out during the academic year 2019-20 at the College of Medicine, King Saud University. The fourth-year undergraduate medical students were the participants. Quantitative data was a self-administered questionnaire and qualitative data was obtained through a focus group discussion (FGD).

### Data Collection & Instrument:

A panel of four experts developed a self-administered questionnaire. Piloting of the survey was done through six faculty members of the department of family medicine. Based on the outcome of the pilot study, 11 items were sorted to achieve the objectives. The survey questionnaire consisted of two sections, a; students’ first experience about FC b; students’ perception of assessment information.

The survey was carried out electronically using the Google Form website and sent via students’ emails (provided by the Student Academic Council). The students participated in the survey anonymously and voluntarily. The students (n= 214) participated in the survey voluntarily.

A total of (n=234) students were enrolled in fourth year. They all were contacted through the student’s email system. Later on, reminder emails were sent to the class leaders to complete the survey.

The convenience sampling method was used to collect qualitative data. An announcement was made in the class about the FG interview, and the names were collected from the interested volunteers. Initially, 22 students enrolled for FG, but on the day of the interview, 15 students arrived. In a two and half hour session, FG discussed the main themes of ‘like,’ ‘dislike,’ and ‘recommendations or suggestions about FC.

### Quantitative data analysis:

The data were coded and analysed using SPSS version 21.0. The associations between the different categorical variables were measured by the Chi-square test statistically, *p*-value <0.05.

### Qualitative data analysis:

FG data were transcribed and analysed using Atlas. ti. A thematic analysis approach was employed. Then coding was done to identify the essential segments of the transcriptions that met the research objectives.

### Ethics approval and consent to participate:

Approval by Institutional Review Board, College of Medicine, King Saud University with reference number 20/0770/IRB.

## RESULTS

A total of 214 students out of 234 [M ;( n=146) 68.2 % and F; (n= 68) 31.8%] completed the survey questionnaire (response rate of 91.45%).

The student’s perceptions about the use of the flipped classroom are summarized (Table-I). The student’s overall responses demonstrated that they agreed the flipped classroom enhanced their learning process of family medicine (mean 2.35±0.36). Majority of students agreed (M±SD = 2.55±0.78); 156 (72.9%) that the flipped classroom was more engaging than the traditional tutor lecture. The correlation between males and females was statistically significant **(**χ2=7.39 *p*=0.02). Furthermore, a very high number of students (79.5% males and 91.1% females) reported improved class communication and discussion with other students before class.

**Table I:** Student’s perceptions about the flipped classroom.

Items	Mean ±SD	Categories†	Participants n (%)	Male n (%)	Female n (%)	χ2 (*P*-value)
The ‘flipped classroom (FC)’ is more engaging than a traditional tutor presented topic?	2.55±0.78	Agree	156(72.9)	100(68.5)	56(82.3)	7.39(0.02)
		Do not know	20(9.3)	13(8.9)	7(10.3)	
		Disagree	38(17.8)	33(22.6)	5(7.3)	
The ‘FC’ gave me more significant opportunities to communicate with other students.	2.70±0.67	Agree	178(83.2)	116(79.5)	62(91.1)	4.72(0.09)
		Do not know	9(4.2)	8(5.5)	1(1.5)	
		Disagree	27(12.6)	22(15.0)	5(7.3)	
The ‘FC’ videos make it easier to understand the materials in advance and work with tutors.	2.56±0.78	Agree	157(73.4)	100(68.5)	57(83.8)	7.56(0.02)
		Do not know	19(8.9)	13(8.9)	6(8.8)	
		Disagree	38(17.8)	33(22.6)	5(7.3)	
The ‘FC’ has helped me learn more than I would have if we had used in-class lectures only.	2.36±0.85	Agree	132(61.7)	82(56.2)	50(73.5)	6.01(0.04)
		Do not know	29(13.6)	22(15.1)	7(10.3)	
		Disagree	53(24.8)	42(28.8)	11(16.2)	
FC makes me more motivated to learn	2.50±0.79	Agree	147(68.7)	90(61.6)	57(83.8)	11.3(0.003
		Do not know	27(12.6)	21(14.4)	6(8.8)	
		Disagree	40(18.7)	35(24)	5(7.4)	
The FC method saves class time to understand a topic.	2.23±0.86	Agree	110(51.4)	79(54.1)	31(45.6)	1.15(0.56)
		Do not know	42(19.6)	27(18.5)	15(22.1)	
		Disagree	62(29)	40(27.4)	22(32.3)	
The FC allows working in a group.	2.69±0.67	Agree	174(81.3)	115(78.8)	59(86.8)	5.34(0.06)
		Do not know	15(7.0)	9(6.2)	6(8.8)	
		Disagree	25(11.7)	22(15.1)	3(4.4)	
I like FC because I can go at my own pace. I don’t have to wait for others.	2.26±0.78	Agree	101(47.2)	74(50.7)	27(39.7)	7.07(0.02)
		Do not know	68(31.8)	38(26)	30(44.1)	
		Disagree	45(21.0)	34(23.3)	11(16.2)	
In the FC method, we get into class we already know the material and the class time is spent clarifying the facts and principles	2.67±0.65	Agree	168(78.5)	108(74)	60(88.2)	5.99(0.05)
		Do not know	23(10.7)	18(12.3)	5(7.4)	
		Disagree	23(10.7)	20(13.7)	3(4.4)	
I do not like the FC method and will not recommend it to my friends.	1.61±0.85	Agree	52(24.3)	40(27.4)	12(17.6)	2.42(0.29)
		Do not know	27(12.6)	18(12.3)	9(13.2)	
		Disagree	135(63.1)	88(60.3)	47(69.1)	
I like the traditional teaching methods more than the FC method.	1.76±0.86	Agree	60(28)	48(32.9)	12(17.6)	5.92(0.05)
		Do not know	44(20.6)	26(17.8)	18(26.5)	
		Disagree	110(51.4)	72(49.3)	38(55.9)	

About the flipped classroom videos, the students (68.5% males and 83.8% females) responded that these helped them understand the class materials in advance and work with tutors. More female students, (83.8%) than male students (61.6%) agreed on more motivation of learning by FC (χ2=11.7, *p*=0.003).

Almost ~79% of students agreed that the most valuable part of the flipped classroom was that when we get to class, we already know the material, and the class time is spent clarifying the facts and principles only. The comparative analysis elucidated that only 27.4% of males and 17.6% of females responded that they did not like the FC and shall ‘not recommend it to friends’ (Table-I).

The student’s perceptions of the course material and assessment of flipped classrooms is summarized in Table-II, which included eight items and an overall Mean±SD 2.34 ±0.13. The majority of the students (74% males and 77.9% females) agreed that it was helpful to do course exercises when other students and tutors were available to answer. Similarly, a higher number of students reported that the flipped classroom saved their time in class, and they had time to raise a subject related question. Likewise, a good number of the students (52.7% of males and 77.9% of females) reported that when they were absent, they watched the course video; they could read PowerPoint slides with references to solve the problem and did not lose the course learning outcomes. Moreover, almost ~ 44% of students (42.5% males and 47.1% females) reported both assessments during the classes, and final assessments were fair, and the difference between the male and female students was statistically significant (*p* =0.001).

**Table II:** Student’s perceptions about flipped classroom course and assessment information.

Items	Mean ±SD	Categories†	Participants n (%)	Male n (%)	Female n (%)	χ2 (*P*-value)
The course modules, including PowerPoint, references, and case, are beneficial in preparing for the class.	2.35±0.88	Agree	135(63.1)	79(54.1)	56(82.4)	15.9(0.000)
		Do not know	21(9.8)	18(12.3)	3(4.4)	
		Disagree	58(27.1)	49(33.6)	9(13.2)	
It is helpful to do course exercises when other students and the professor are available to answer questions as opposed to doing the homework exercises alone	2.51±0.63	Agree	161(75.2)	108(74)	53(77.9)	0.44(0.80)
		Do not know	34(15.9)	24(16.4)	10(14.7)	
		Disagree	19(8.9)	14(9.6)	5(7.4)	
It saves time in class because I have more time to ask questions if I don’t understand	2.51±0.79	Agree	151(70.6)	97(66.4)	54(79.4)	4.0(0.13)
		Do not know	23(10.7)	17(11.6)	6(8.8)	
		Disagree	40(18.7)	32(21.9)	8(11.8)	
I have liked how quick and easy it is to learn by reviewing PowerPoint slides with references.	2.18±0.90	Agree	111(51.9)	67(45.9)	44(64.7)	19.0(0.000)
		Do not know	31(14.5)	16(11)	15(22.1)	
		Disagree	72(33.6)	63(43.1)	9(13.2)	
I liked it because when you absent, you still can watch videos, PowerPoints slides with references to solve the problem and not get behind	2.37±0.83	Agree	130(60.7)	77(52.7)	53(77.9)	13.0(0.001)
		Do not know	35(16.4)	27(18.5)	8(11.8)	
		Disagree	49(22.9)	42(28.8)	7(10.3)	
Assessment of students through flipped classroom was fair and very educational	2.27±0.76	Agree	100(46.7)	65(44.5)	35(51.5)	11.7(0.003)
		Do not know	73(34.1)	44(30.1)	29(42.6)	
		Disagree	41(19.2)	37(25.3)	4(5.9)	
Both continue assessment during the classes, and final assessment was fair.	2.16±0.83	Agree	94(43.9)	62(42.5)	32(47.1)	14.8(0.001)
		Do not know	61(28.5)	33(22.6)	28(41.2)	
		Disagree	59(27.6)	51(34.9)	8(11.8)	
Do you think data interpretation was beneficial?	2.44±0.82	Agree	141(65.9)	91(62.3)	50(73.5)	5.61(0.06)
		Do not know	27(12.6)	17(11.6)	10(14.7)	
		Disagree	46(21.5)	38(26)	8(11.8)	

### Qualitative analysis:

***Stage one*-** students liked the FC, and they began to recognize what would be taught during an FC. In the FC, students had better opportunities to ask questions from their peers and or tutors.

### Better opportunities for asking the questions and answers:

Some of the responses of Focus Group (FG) are highlighted as: *“More chance to discussing with the doctors, and I got the chance to answer”* (FG#6). *“When I discuss with doctors and other students, I get more interesting information in the days before the final, it was easy to study more than in traditional methods because I know almost everything important in each topic.”* (FG#2).

The students found that videos were useful for learning clinical skills. *“I think the videos help a lot. Watching videos about the information like simulation, you will [make you] remember”* (FG#14).

### Improved group discussion:

Discussion is an essential process of flipped class to reach a higher level of comprehension. One student mentioned *“Somewhere, I get confused and not understand the video, so I ask the question, my friends and teacher can answer and explain to me clearly.”* (FG#1). Students also mentioned peers as a reliable source of reference. Few students testified*”The best thing about the flipped class I will study with a group if I finish only in a few sessions, I review the entire group.”* (FG#15) *“You can have it [study group] as a revision session to discussed something you don’t understand to just have it like, especially in the clinical exam”* (FG#8)

***Stage two-***students disliked the FC, where they began to identify the difficulty in the flipped classroom,

### Shortage of time:

Some students reported that the FC sessions were short, *“I think the only thing I dislike is the shortness of time”* (FG# 2, 6, 4, 11). *“Not dislike, but we hope the (materials) send to us at the begging of the course.”* (FGD#15) mentioned. Some students also considered that there were some issues (not useful) regarding FC, e.g., *“In the flipped classroom, there was no clinical rotation at the hospital; the patients were simulated in videos; I’d prefer to have a learning experience from actual patients in the clinic.”* (FG#14, 5, 8). *“Not real patient, very long references for some topics, not enough illustrated information in PowerPoint slides.”* (FG#6)

### Stage three- Suggestions: Videos, notes, and slides should be goals oriented:

Most students seemed to watch the videos once as we asked them to but would usually not watch them again for the review. Furthermore, some videos were not related to specific course objectives *“Maybe if the topic presentation was organized, it becomes easier to understand* *if you only give us more specific objective related video references for each point in the topics, it would be better, and I try to connect to review the videos, but I could not”* (FG#9). Two students reported an almost similar problem *“I like the flipped classroom, but some slides animation was not good”* (FG#4, 13).

## DISCUSSION

In traditional classroom approaches, fast learner students have to stay with relatively slow learners, as the tutor might have to repeat the topic. In the FC environment, students adjust the time of learning at their own pace and learn the new subject independently or within a group outside the class hours. Our study also reported that most students like the FC more than the traditional teaching methods during the pandemic period. There was a lockdown situation and everybody had to stay at home. The same findings were reported in another study published in the United States.^9^ Students could access the video lesson everywhere in suited to their comfort places such as classroom, coffee shop, park, or home and they could use several devices to watch the flipped classroom videos such as a laptop, computer, or Smartphone.^10^,^11^

In this study ~71% of students agreed that the flipped classroom saved time and they were not feeling any pressure to ask a question any time; the same observation was reported where students have more time to watch videos whenever they want without feeling any pressures of time and asking more question to instructors.^12^ The current study reported the availability of extra time for students to interact with classmates or instructors through group discussion, which enhanced higher-order thinking skills. Besides, most students recognized that flipped classroom teaching supported the establishment of good communication with peers. The result was similar to a previously published study.^13^

The findings show that flipped classrooms allow for better communication, helping them to know the topic and material in advance, and they could clarify the facts in the discussion session. In two recently published studies, Tomas et al.^14^ and Angadi et al.^15^ argue that it is still beneficial to employ a task-oriented environment supported by a well-designed structure that guides students to solve given problems.

A clear course structure with supporting tools such as guiding prompts and instructions must be designed to help students prepare for participation to achieve learning goals. The findings also show issues such as a few videos were slow and not relating to the specified objectives and ‘some videos only contained spoken verbatim without animation. This suggests that flipped classroom videos are still at an early stage of development. But students made few good suggestions to improve the future flipped classroom, especially in the Saudi context, e.g., divide the lecture time and presentation time, and a lot of room for improvement, particularly in the multimedia proportion. The future design of flipped classroom video lessons must be concerning the course content and situated in a particular spot or position in an authentic context.

Education needs a variety of instruction methods that may enable and encourage self-directed learning. This study searched a learner-focused approach in a medical course to improve student’s motives and enable them towards the usage of technology and advanced techniques. After the successful implementation of the FC, the current study was designed to judge its effectiveness in comparison to a traditional classroom for the Family Medicine course. In the present study, the findings match with a previous study^15^ where students were found confused about the type of method used for learning activities in an FC. The students had constructed their communication, understanding of materials, discussion engagement in-class activities. This statement is like the previously published findings where students were more positive and active in classroom activities because they come to class prepared before any discussion started.^16^,^17^ Concurring with previously published studies on the flipped classroom, students want to study material (flipped classroom videos) in advance to understand the topic.^18^,^19^ The students like the flipped classroom more than the traditional style to learn a section from the videos was also agreeing with a previous study.^20^,^21^

### Limitation of the study and further recommendation:

This study was Unicenter. We encourage other researchers to engage the data for multiple disciplines to consolidate the results especially from the aspect of Non-Face-to-Face learning that is an effective and influential tool in the calculation of credit hours. 22 Also, one discipline was considered to receive the perception on FC modality during the COVID-19 period. Lacking the faculty perspective was also a limitation. There is a need for faculty to be trained^23^ before it is implemented especially during COVID-19 and the role of medical educator gets immense and extensive.

## CONCLUSION

Sharing the experiences and observation of transforming the traditional classroom into the Flipped Classroom for undergraduate medical students showed that FC was more likely to be an effective teaching-learning mode. The implementation of FC is a shared responsibility between students and instructors and it may accelerate academic excellence. There should be more FC sessions with other topics of medicine. A group of instructors may be dedicated to developing videos with the help of the simulation department. Some suggestions to identify the problems of students understanding were also laid out with the expectation of improved active learning. However, there is still room for improvement on flipped classrooms in future. We recommend that future research be involved in large sample sizes, incudes more subjects, and different years of perception. The benefits of FC are robust and likely to augment the learning abilities of the students as well as supplementing the learning course content; group events can deliver added benefits too.
